# Variational Model for Single-Image Reflection Suppression Based on Multiscale Thresholding

**DOI:** 10.3390/s22062271

**Published:** 2022-03-15

**Authors:** Pei-Chiang Shao

**Affiliations:** Department of Mathematics, Soochow University, Taipei City 111002, Taiwan; shaopj823@gmail.com; Tel.: +886-2-2881-9471 (ext. 6709)

**Keywords:** multiscale thresholding, reflection removal, reflection suppression, variational model

## Abstract

Reflections often cause degradation in image quality for pictures taken through glass medium. Removing the undesired reflections is becoming increasingly important. For human vision, it can produce much more pleasing results for multimedia applications. For machine vision, it can benefit various applications such as image segmentation and classification. Reflection removal is itself a highly illposed inverse problem that is very difficult to solve, especially for a single input image. Existing methods mainly rely on various prior information and assumptions to alleviate the ill-posedness. In this paper, we design a variational model based on multiscale hard thresholding to both effectively and efficiently suppress image reflections. A direct solver using the discrete cosine transform for implementing the proposed variational model is also provided. Both synthetic and real glass images are used in the numerical experiments to compare the performance of the proposed algorithm with other representative algorithms. The experimental results show the superiority of our algorithm over the previous ones.

## 1. Introduction

For most multimedia and computer vision applications, the input images are normally assumed to be both clean and clear. However, bad lighting conditions and environments such as photographing behind a window glass or showcase are almost inevitable in our daily life. In these scenarios, the captured images often contain undesired reflections from the objects on the camera side, which severely degrade image quality and affect the performance of multimedia and computer vision applications, such as image segmentation and classification.

Due to the rapid growth of mobile devices, a fast and effective reflection removal algorithm is increasingly desired. Reflection removal is the restoration process that aims essentially at removing or suppressing unpleasing reflections and improving the visibility of the scenes in front of the glass medium. As a preprocessing step, it can benefit various follow-up applications.

Barrow et al. [1] first proposed a linear assumption for an observed degraded image *Y* as a superposition of two layers as follows:

(1) Y=T+R,

where *T* and *R* are the transmission and reflection layers, respectively. The inverse problem of resolving *T* and *R* given *Y* is highly ill-posed, since there is only one equation for two unknowns per pixel. According to the number of input images *Y* used, reflection removal methods can be classified into multiple-image and single-image approaches, where the former makes the problem less ill-posed while the latter is itself highly ill-posed and challenging. Since perfectly separating the transmission and reflection layers is, in general, very difficult even for the case of multiple input images and, in most situations, people tend to care only about the transmission layers, estimating a reasonable *T* that might contain slightly misclassified information from *R* can be considered instead. To make the ill-posed problem more tractable for single input image, researchers tried to suppress the reflection in the image (i.e., estimating a reasonable *T*) instead of removing the reflection (i.e., solving an exact separation of *T* and *R*) [2,3]. In this paper, we follow the same idea and consider the reflection suppression problem instead.

Multiple-image approaches generally use several degraded images as input, which are captured by a fixed camera. Kong et al. [4] employed a physical formation model and used different angular filters to estimate the optimal reflection layer. Similarly, Schechner et al. [5] focused on various distances using multiple images for reflection removal. Farid and Adelson [6] employed independent component analysis to separate reflections from glossy surfaces or glass using two images with different polarization angles. Agrawal et al. [7] made use of two degraded images captured with and without a flash exposure to identify reflections. Multiple images captured with different camera positions were also considered. Sirinukulwattana et al. [8] exploited varying reflections taken from different viewpoints to smooth reflections and enhance the transmission. Similarly, Li and Brown [9] exploited the subtle changes in the reflection via different viewpoints. Video sequences were also used to separate transmission and reflection layers. Xue et al. [10] exploited motion differences and recovered the transmission and reflection layers. Gai et al. [11] jointly estimated layer motions via a blind separation algorithm. Guo et al. [12] exploited the correlation of the transmission layers across multiple images to separate the two layers. Multiple input images largely reduce the ill-posedness of the reflection removal problem. However, due to fastidious image acquisition requirements, algorithms based on multiple images have limited practical applications.

In most situations, one may not have the chance to capture multiple images of the same scene, which is especially difficult with slightly different camera positions or polarization angles. As a consequence, single-image approaches are becoming practically important. As mentioned above, reflection removal based on a single input image is itself a highly ill-posed problem, and existing methods mainly assume different image priors for the transmission and reflection layers. Levin and Weiss [13] exploited sparse gradient priors with manual labels to distinguish the two layers. Li and Brown [14] assumed that two layers have different smoothness and imposed the smooth and sparse gradient priors over the two layers. Shih et al. [15] exploited the Gaussian mixture model prior for the reflections to solve a deblur optimization problem. Wan et al. [16] exploited the depth of field in a multiscale manner using K-L divergence to identity edges of different layers.

Recently, deep neural networks are also adopted to remove reflection. Fan et al. [17] proposed the first deep neural network to solve the layer separation problem. They exploited edge information to guide the separation process. Wan et al. [18] integrated a gradient inference network with a image inference network as a concurrent network to remove the reflection of a single input image. Yang et al. [19] proposed a cascade deep network to bidirectionally estimate the transmission and reflection layers. More precisely, the network uses for the estimated transmission to estimate reflection and then useds for the estimated reflection to estimate transmission. Zhang et al. [20] proposed a convolutional network with a feature loss, an adversarial loss, and an exclusion loss that enforces the layer separation. The main advantage of deep learning-based approaches for reflection removal is that no handcrafted image priors are necessary. However, a large amount of deep network training time and training datasets is required.

The remainder of this paper is organized as follows. In Section 2, we briefly review two related works for reflection suppression. In Section 3, we present the improved reflection suppression model for a single-input image. The proposed model is based on multiscale hard thresholding, and a direct solver using the discrete cosine transform for implementing the proposed model is also given in Section 3. Numerical experiments are conducted in Section 4 to demonstrate the superior performance of the newly proposed model. Finally, some concluding remarks and discussions are given in Section 5.

## 2. Related Work

As introduced in Section 1, reflection removal is a highly ill-posed inverse problem that is very challenging to solve, especially for the case of a single input image. Arvanitopoulos et al. [2] tried to suppress the reflection instead of removing the reflection. They exploited a Laplacian data fidelity term and a sparse gradient prior, which achieve satisfactory quality for reflection suppression. However, since their model is nonconvex, their algorithms are quite inefficient and require a large number of iterations to converge to a desirable result.

As an improvement of [2], Yang et al. [3] proposed a hard thresholding operation to the gradient of the input image in the Laplacian data fidelity term. Their method also achieves satisfactory quality for reflection suppression, but the algorithm is highly efficient compared with [2]. Since our method is an improvement of [3], we briefly review the works of [2,3] in the following two subsections.

### 2.1. Smooth Regularization on T

The algorithm proposed in [2] for reflection suppression mainly relies on the critical assumption that reflection edges (gradients) are smaller in magnitude and less in focus compared to transmission edges. This assumption is reasonable in real-world scenarios since the camera focuses on transmission objects rather than reflection components. Mathematically, this assumption can be expressed as the following formation model [21]:

(2) $$Y=W\circ T+\left(1-W\right)\circ \left(k_{\sigma }*R\right),$$

where ∘ and ∗ denote the component-wise multiplication and the convolution operation, respectively. *W* is the contribution weight of the transmission *T* to the observation *Y*, and $k_{\sigma }$ is a Gaussian blur kernel with standard deviation σ performed on the reflection layer *R*. In general, *W* is pixel-dependent and depends on the lighting conditions and the distance of the camera to the captured scene. For simplicity, weight *W* can be considered as a constant, i.e., W(x)=w,∀x∈Ω, where Ω and $x=(x_{1},x_{2})$ denote the image domain and a pixel point, respectively.

Following the successful smoothing model of Xu et al. [22], Arvanitopoulos et al. [2] employed the number of non-zero gradients as the prior information to restrict and regularized output transmission. Their model can eliminate small gradients and simultaneously preserve large image edges. The original minimization problem in [22] is as follows:

(3) $$\min_{T}{∥T-Y∥}_{2}^{2}+\lambda C\left(T\right),$$

where the following:

(4) $${∥T-Y∥}_{2}^{2}:=\min_{T}\int_{\Omega }{\left(T-Y\right)}^{2}\;dx,$$

is the $L^{2}$ data fidelity term which keeps the optimal *T* close to the observation *Y*, and the following:

(5) C(T):=♯{x∈Ω:|∇T(x)|≠0},

is the $L^{0}$ regularization term where ♯ denotes the cardinality measure applied on a given set (i.e., a measure of the number of elements of the set). The $L^{0}$ term regularizes the optimal *T* by implicitly performing a hard thresholding of its gradients with threshold λ. Since high frequency details of the image will be eliminated, model (3) is suitable for image smoothing but unsuitable for reflection suppression.

To preserve high frequency details in optimal *T*, Arvanitopoulos et al. [2] proposed a Laplacian data fidelity term to replace (4), where the Laplacian of the optimal *T* is defined by the following.

(6) $$L\left(T\right):=\mathrm{div}\left(\nabla T\right)=\frac{\partial^{2}\;T}{\partial x_{1}^{2}}+\frac{\partial^{2}\;T}{\partial x_{2}^{2}}.$$

Their data fidelity term based on the Laplacian to enforce the consistency in fine structures is then given as follows:

(7) $$\min_{T}{∥L\left(T\right)-L\left(Y\right)∥}_{2}^{2}+\lambda C\left(T\right),$$

where regularization term C(T) is the same as defined in (5). The new model can preserve strong edges and details in transmission *T* while eliminating reflection *R* simultaneously; thus, it is more suitable for reflection suppression.

### 2.2. Direct Thresholding on Y

In [2], the edge information of the optimal *T* is preserved by applying the Laplacian differential operator L. The reflection component *R* is suppressed by applying regularization *C* on *T*. As the regularization parameter λ increases, more reflections are suppressed in *T*. However, as their model defined in (7) is nonconvex, their operator splitting algorithm for minimizing the nonconvex energy functional is highly inefficient and takes a large number of iterations to converge to a desired solution. Therefore, Yang et al. [3] integrated these ideas into a new model formulation, which significantly reduces computation times while retaining satisfactory reflection suppression performances.

Instead of using the hard thresholding operation derived implicitly from the smoothing regularizer *C* on *T*, Yang et al. [3] adopted the idea from [23,24] to directly integrate the hard thresholding operation for gradients on *Y* into the energy functional, which is expressed as follows:

(8) $$\min_{T}\frac{1}{2}∥L\left(T\right)-\mathrm{div}\left(\delta_{h}\left(\nabla Y\right)\right){∥}_{2}^{2}+\frac{\varepsilon }{2}{∥T-Y∥}_{2}^{2},$$

where $\delta_{h}$ is the hard thresholding operation for gradients and is defined as follows.

(9) $$\delta_{h}\left(G\left(x\right)\right):=\left\{\begin{array}{cc} G(x), & if\;|G(x)|\geq h\\ 0, & if\;|G(x)|<h \end{array}\right..$$

In the first term of (8), the gradient of the observation *Y*, i.e., ∇Y, is thresholded using $\delta_{h}$ before taking the divergence operator div. Since ∇Y(x) with magnitude |∇Y(x)| smaller than *h* will be truncated to zero, the model has the ability to suppress reflections in *T*. Since the data term also enforces the consistency of *T* to *Y* via Laplacian operator L, fine details of *Y* will be preserved simultaneously in *T*. Therefore, the $\delta_{h}$ in (8) takes over the role of *C* in (7) for reflection suppression.

Since the data fidelity term in (8) contains only second-order derivatives of transmission *T*, the optimal solution of *T* will not be unique, and any optimal transmission *T* shifted by a affine function *A*, i.e., T+A, will also be an optimal transmission. To ensure the uniqueness of the optimal solution, the second term of (8) is incorporated. The model parameter ε should be taken as a positive tiny number so as not to cancel out the effect of reflection suppression, which is performed via the first term.

## 3. Proposed Method

In this section, we present an improved version of the model of Yang et al. [3] given in (8) for single-image reflection suppression. The proposed model is based on multiscale hard thresholding, and a direct solver using the discrete cosine transform for implementing the proposed model is also given.

### 3.1. Multiscale Thresholding

The hard thresholding model given in (8) suppresses the reflections by eliminating the gradients of *Y* at pixel point *x* with magnitude |∇Y(x)| less than the prescribed threshold value *h*. For a small *h*, the model eliminates only small gradients; thus, only weak reflections will be suppressed while strong reflections remain in the optimal transmission. For a large *h*, the model eliminates both small and large gradients; thus, both weak and strong reflections will be suppressed simultaneously. However, when *h* is large, many important image edges in the transmission with their gradient magnitudes smaller than *h* will also be suppressed. Therefore, a large *h* in (8) usually causes information loss in the optimal transmission. This is the reason why the model should assume that reflection edges are smaller in magnitude and less in focus compared to transmission edges. If this assumption fail to hold, the model will produce either (1) a blurry transmission without any reflections when *h* is large or (2) a clear transmission but with many reflections remaining when *h* is small. Therefore, a moderate *h* is normally picked to trade off between situations (1) and (2) to obtain an acceptable performance.

To suppress both weak and strong reflections while preventing the information loss of important edges in the optimal transmission, we propose a multiscale hard thresholding energy functional to improve the performance of the model of Yang et al. in (8). Beginning with the smallest thresholding scale *h*, our model additionally considers another N−1 countable thresholding scales, i.e., {2h,3h,…,Nh}. Hence, there are totally *N* scales in our model, that is, {h,2h,3h,…,Nh}, where scale number *N* is a prescribed positive integer which can be determined by the user. The model in (8) is first modified as follows.

(10) $$\min_{T}\frac{1}{2}\sum_{n=1}^{N}∥L\left(T\right)-\mathrm{div}\left(\delta_{nh}\left(\nabla Y\right)\right){∥}_{2}^{2}+\frac{\varepsilon }{2}{∥T-Y∥}_{2}^{2}.$$

When N=1, the multiscale energy functional reduces to the energy functional of Yang et al. in (8). When N≥2, the multiscale energy functional has N+1 terms, and the first *N* terms are linearly fused into one with uniform weights $\frac{1}{2}$, where each term involves a hard thresholding operation on ∇Y with threshold value nh at *n*-th scale. Therefore, the first term in (10) can be viewed as a fusion term to integrate all information of the thresholded Laplacian of *Y* at different scales. As *n* increases, more and more weak edges are eliminated; thus, only strong edges are left. In other words, the small *n* term provides the suboptimal transmission *T* with both strong and weak edges while the large *n* term provides the suboptimal transmission *T* with only strong edges. After adding the second term in (10) to ensure the uniqueness of the optimal solution, the final optimal transmission *T* can be viewed as a linear combination of the sub-optimal transmissions of those subfunctionals corresponding to different thresholding scales. The model parameter ε is also set to be a positive tiny number just as that provided in (8). In this paper, ε will be fixed at $10^{-6}$.

To further enhance the preserved edge information, we proposed a pixel-dependent weight function φ to adapt the thresholded gradients pixelwisely for all subfunctionals in (10) to form our final model for reflection suppression:

(11) $$\min_{T}\frac{1}{2}\sum_{n=1}^{N}∥L\left(T\right)-\mathrm{div}\left(\varphi \delta_{nh}\left(\nabla Y\right)\right){∥}_{2}^{2}+\frac{\varepsilon }{2}{∥T-Y∥}_{2}^{2},$$

where the adaptive weight function φ is designed to be inversely proportional to |∇Y| as follows.

(12) $$\varphi \left(x\right)=\left(1-\frac{\left|\nabla Y(x)\right|}{\max_{x\in \Omega }\left|\nabla Y\left(x\right)\right|}\right)+\beta ,\;\;\forall x\in \Omega .$$

The role of shifting β in (12) is to maintain a base level of the adaptive weight function. In this paper, β will be fixed at 1.0.

### 3.2. Solving the Model

Since the energy functional proposed in (11) is convex, a transmission *T* is the optimal solution to (11) if and only if it satisfies the Euler-Lagrange equation derived step by step below:

(13) $$\begin{array}{cc} \; & L^{*}\left(\sum_{n=1}^{N}\left(L\left(T\right)-\mathrm{div}\left(\varphi \delta_{nh}\left(\nabla Y\right)\right)\right)\right)+\varepsilon \left(T-Y\right)=0\\ \Rightarrow & \left(\sum_{n=1}^{N}L^{*}L\left(T\right)\right)+\varepsilon T=L^{*}\left(\sum_{n=1}^{N}\mathrm{div}\left(\varphi \delta_{nh}\left(\nabla Y\right)\right)\right)+\varepsilon Y\\ \Rightarrow & \left(NL^{*}L+\varepsilon \right)T=L^{*}\left(\sum_{n=1}^{N}\mathrm{div}\left(\varphi \delta_{nh}\left(\nabla Y\right)\right)\right)+\varepsilon Y\\ \Rightarrow & \left(NLL+\varepsilon \right)T=L(\sum_{n=1}^{N}\mathrm{div}\left(\varphi \delta_{nh}\left(\nabla Y\right)\right))+\varepsilon Y \end{array}$$

where $L^{*}$ denotes the adjoint operator of L. Since L is self-adjoint, we have $L^{*}=L$. Taking the Fourier cosine transform $F_{c}$ on both sides, we have the following.

(14) $$\begin{array}{cc} \Rightarrow & F_{c}\left[\left(NLL+\varepsilon \right)T\right]=F_{c}\left[L\left(\sum_{n=1}^{N}\mathrm{div}\left(\varphi \delta_{nh}\left(\nabla Y\right)\right)\right)+\varepsilon Y\right]\\ \Rightarrow & \left(NF_{c}\left(L\right)\circ F_{c}\left(L\right)+\varepsilon \right)\circ F_{c}\left(T\right)=F_{c}\left[L\left(\sum_{n=1}^{N}\mathrm{div}\left(\varphi \delta_{nh}\left(\nabla Y\right)\right)\right)+\varepsilon Y\right] \end{array}$$

Denoting $K=F_{c}\left(L\right)$ and using component-wise division, we have the following.

(15) $$F_{c}\left(T\right)=\frac{F_{c}\left[L\left(\sum_{n=1}^{N}\mathrm{div}\left(\varphi \delta_{nh}\left(\nabla Y\right)\right)\right)+\varepsilon Y\right]}{NK\circ K+\varepsilon }$$

Taking the inverse Fourier cosine transform $F_{c}^{-1}$, we arrive at the optimal transmission of (11) as follows:

(16) $$T=F_{c}^{-1}\left[\frac{F_{c}\left[L\left(\sum_{n=1}^{N}\mathrm{div}\left(\varphi \delta_{nh}\left(\nabla Y\right)\right)\right)+\varepsilon Y\right]}{NK\circ K+\varepsilon }\right],$$

where *K* is the Fourier cosine transform of the Laplacian operator, which can be represented, in its finite dimensional matrix form on an I×J digital image lattice, as follows:

(17) $$K_{i,j}=2\left(\cos\left(\frac{i\pi }{I}\right)+\cos\left(\frac{j\pi }{J}\right)-2\right),\;\;0\leq i\leq I-1,\;0\leq j\leq J-1,$$

and the differential operators L, div and *∇* can be replaced with standard finite difference schemes [25] for digital input images $\left[Y_{i,j}\right]$ for 0≤i≤I−1 and 0≤j≤J−1.

**Figure 1:**
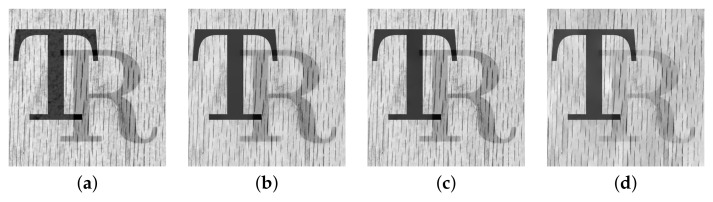
Comparison using a toy example. (**a**) The input test image. (**b**) The result of algorithm [2]. (**c**) The result of algorithm [3]. (**d**) The result of proposed algorithm with N=4.

The overall algorithm of the proposed multiscale thresholding method for reflection suppression is summarized in (Algorithm 1), and a toy example in Figure 1 shows the effectiveness of the proposed algorithm.
**Algorithm 1:** The proposed algorithm for reflection suppression by multiscale thresholding 
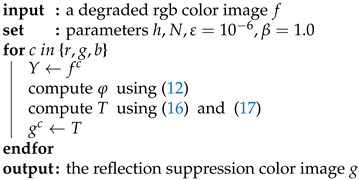


## 4. Numerical Experiment

In this section, we compare the proposed algorithm with the reflection suppression algorithms of Arvanitopoulos et al. [2] and Yang et al. [3], which are all implemented in MATLAB. Both synthetic and real glass images are tested. All experiments are implemented using MATLAB 2019a executed on a Windows PC with Intel Core i7-4790 processor and 16 GB RAM.

### 4.1. Synthetic Glass Image

**Figure 2:**
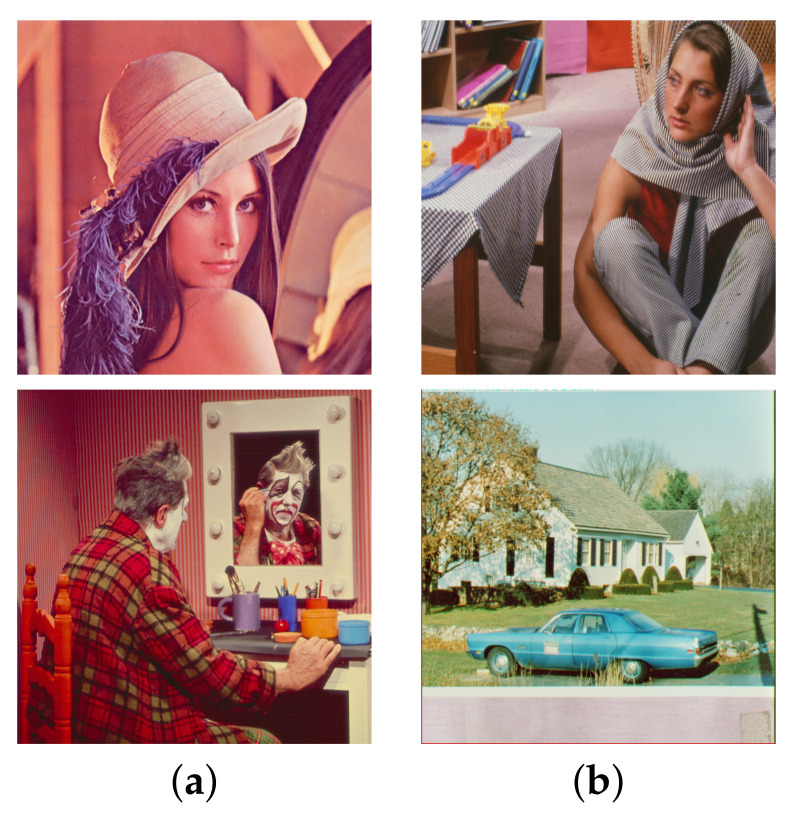
Two pairs of images to be synthesized. (**a**) Two transmission images: ‘Lena’ and ‘Clown’. (**b**) Two reflection images: ‘Barbara’ and ‘House’. The sizes of these four images are all 512×512.

Two pairs of images of size 512×512 were blended into one using Equation (2) to synthesize the test images. The constant weight $W_{i,j}\equiv w$ is set at three different values, which are 0.7, 0.6, and 0.5. The standard deviation σ=2 is used for the Gaussian blur kernel. The model parameters are fixed at λ=0.002 for algorithm [2] and h=0.01, $\varepsilon =10^{-6}$, for both algorithm [3] and the proposed algorithm according to the suggestions in [2,3]. Figure 2a shows two transmission images ‘Lena’ and ‘Clown’ and Figure 2b shows two reflection images ‘Barbara’ and ‘House’. The first test image was synthesized by blending ‘Lena’ with ‘Barbara’ using constant weight *w*, and the second test image was synthesized by blending ‘Clown’ with ‘House’ using constant weight *w*.

**Figure 3:**
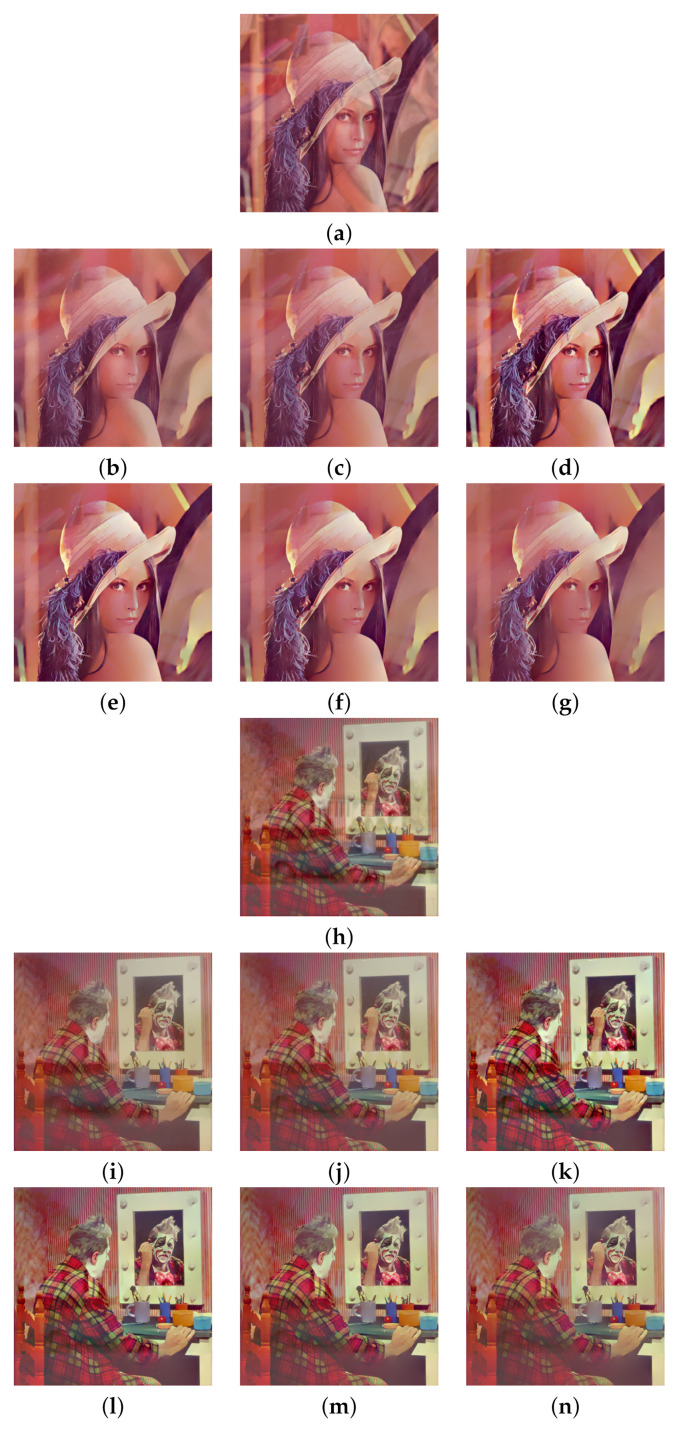
Reflection suppression results for synthetic glass images with w=0.7. (**a**,**h**) Input images. (**b**,**i**) The results of algorithm [2]. (**c**,**j**) The results of algorithm [3]. (**d**,**k**), (**e**,**l**), (**f**,**m**), and (**g**,**n**) are results of the proposed algorithm with N=1,2,3,4, respectively.

We begin with w=0.7, which corresponds to a relatively weak reflection effect among the three cases. Figure 3 shows the reflection suppression results of all competing algorithms for the two synthetic glass images. It can be found by observing Figure 3d,k that the proposed algorithm with scale number N=1 simultaneously possesses very significant image enhancement effects than the two competing algorithms in Figure 3b,c,i,j. As the scale number *N* increases from 2 to 4 as Figure 3e–g,l–n demonstrates, the proposed algorithm is able to suppress more and more reflections while preserving the edge structures of the transmission images.

**Figure 4:**
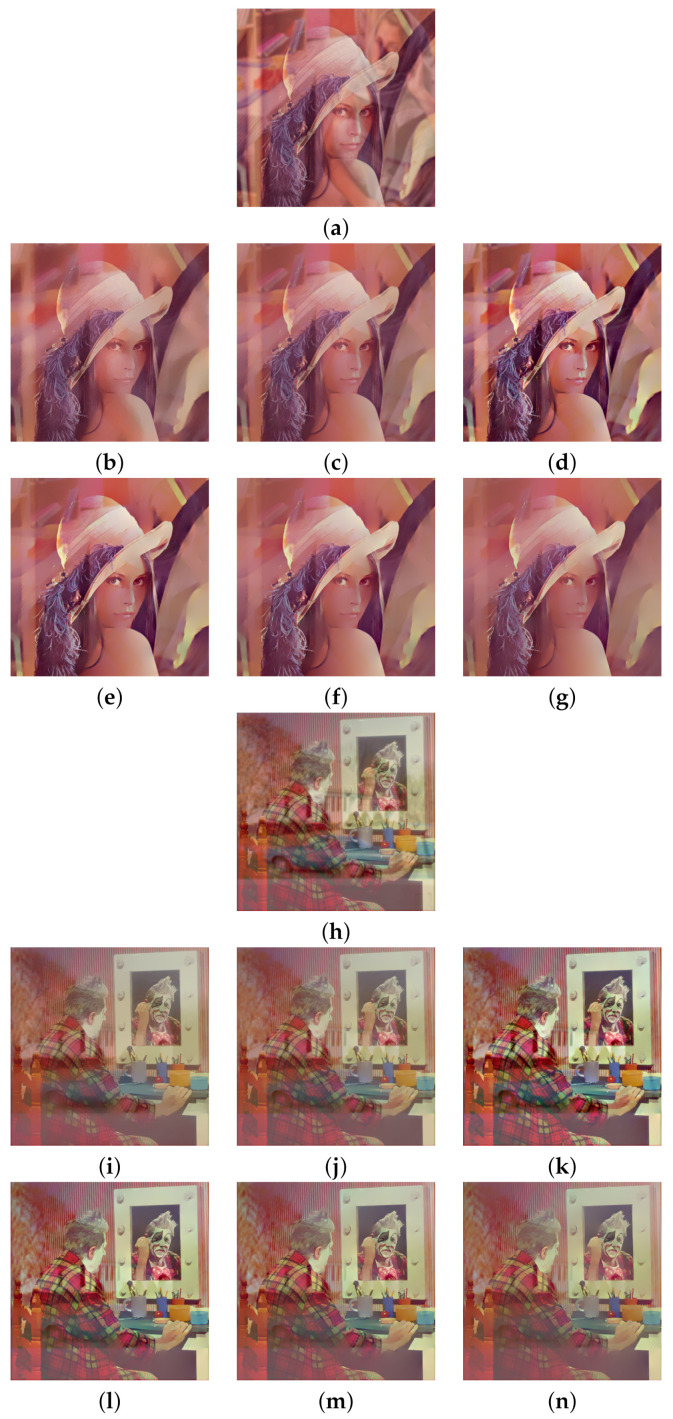
Reflection suppression results for synthetic glass images with w=0.6. (**a**,**h**) Input images. (**b**,**i**) The results of algorithm [2]. (**c**,**j**) The results of algorithm [3]. (**d**,**k**), (**e**,**l**), (**f**,**m**), and (**g**,**n**) are the results of the proposed algorithm with N=1,2,3,4, respectively.

**Figure 5:**
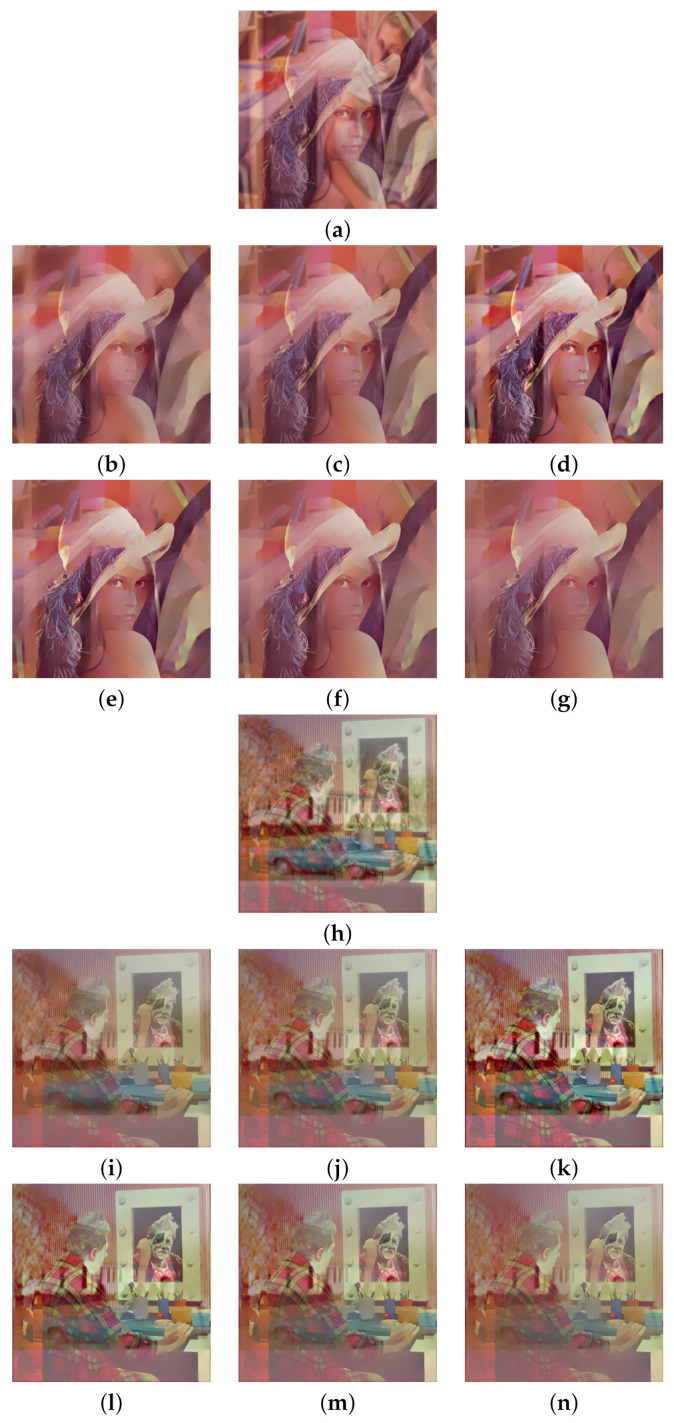
Reflection suppression results for synthetic glass images with w=0.5. (**a**,**h**) Input images. (**b**,**i**) The results of algorithm [2]. (**c**,**j**) The results of algorithm [3]. (**d**,**k**), (**e**,**l**), (**f**,**m**), and (**g**,**n**) are results of the proposed algorithm with N=1,2,3,4, respectively.

Similar results can be found in Figure 4 and Figure 5, which correspond to the relatively moderate (w=0.6) and strong (w=0.5) reflection effects, respectively. The proposed algorithm with N=1 in Figure 4d,k and Figure 5d,k still show significant enhancement effects and the reflections are increasingly suppressed as *N* increases. To evaluate the competing algorithms quantitatively, we compute the PSNR value for each reflection suppression result using the underlying true transmission image as the reference image. Table 1 shows the PSNR values, and the best value in each row is shown in boldface. It can be seen that the proposed algorithm with N=2 has the best PSNR values in all cases, and the PSNR values for N=3 and N=4 keep very close to that of algorithms [2,3]. This shows that the proposed algorithm is superior to the two representative algorithms. Theoretically, a large *N* in (11) can suppress more reflections but obtains lower PSNR values since important edges of the transmission are lost in the same time. On the other hand, a small *N* in (11) can get higher PSNR values but suppressing less reflections. Empirical observations from Table 1 suggest that one can set N=2 for high PSNR values or set N=4 for suppressing more reflections. N=3 is another choice that balances these two extreme choices.

We note that algorithms [2,3] produce very similar image quality and PSNR values. However, this is an expected phenomenon. Although the smooth regularization on *T* in (7) and the direct thresholding on *Y* in (8) have quite different functional forms, their core ideas are rather similar, i.e., “to eliminate small image gradients”. Model (8) directly eliminates the image gradients of *Y*, which are less than *h* in magnitude. For small *h*, less image gradients are eliminated; for large *h*, more image gradients are eliminated. On the other hand, model (7) eliminates the image gradients of *T* in the order of small magnitude to large magnitude, and the number of total eliminated gradients will be determined by model parameter λ. For small λ, less image gradients are eliminated; for large λ, more image gradients are eliminated. Although tuning parameters *h* and λ can produce very similar results and quality, they are still different since the energy functionals, and (7) and (8) are not equivalent. The two main advantages of model (8) compared to model (7) are as follows: (i) tuning the parameter *h* is much more intuitive than tuning the parameter λ since *h* is directly related to gradient magnitude while λ is not; and (ii) the energy functional (8) is convex in *T* while the energy functional (7) is nonconvex in *T*, which means that their algorithm will be less efficient.

### 4.2. Real Glass Image

**Figure 6:**
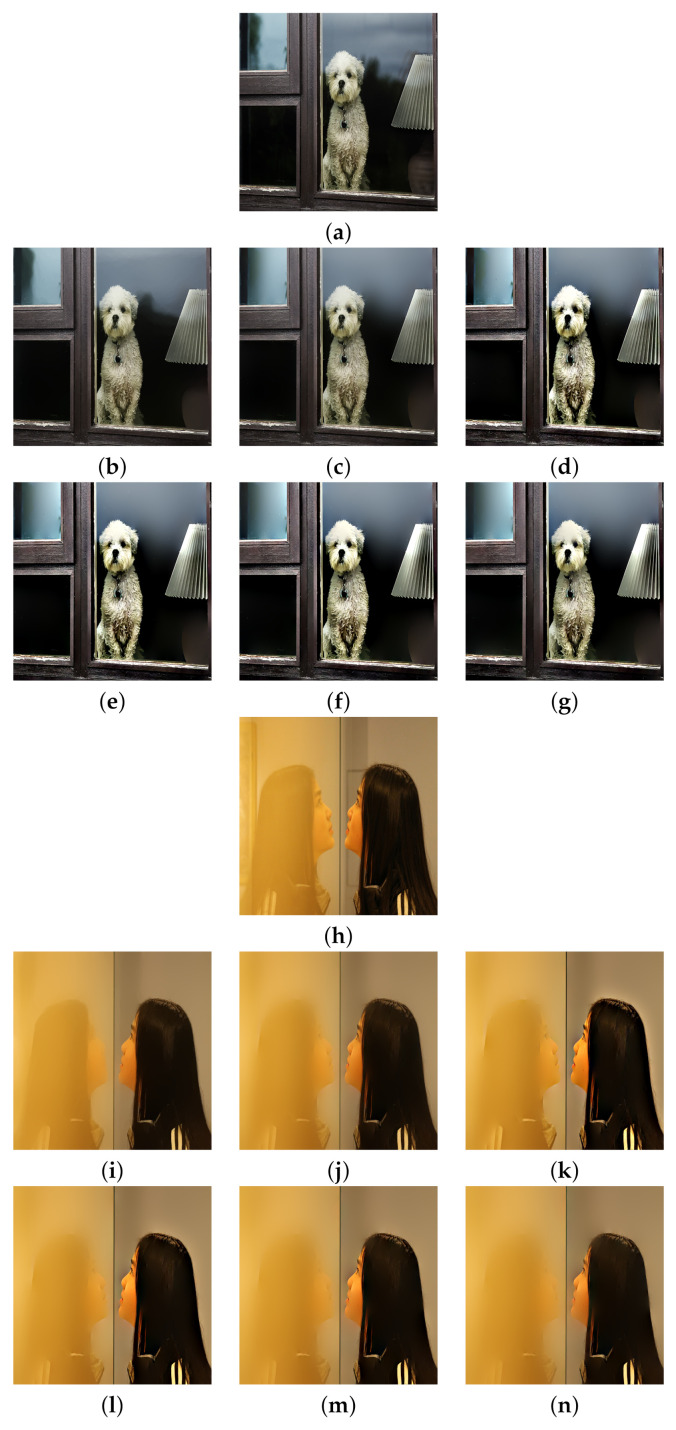
Reflection suppression results for real glass images ‘Dog’ and ‘Girl’. (**a**,**h**) Input images. (**b**,**i**) The results of algorithm [2]. (**c**,**j**) The results of algorithm [3]. (**d**,**k**), (**e**,**l**), (**f**,**m**), and (**g**,**n**) are results of the proposed algorithm with N=1,2,3,4, respectively.

**Figure 7:**
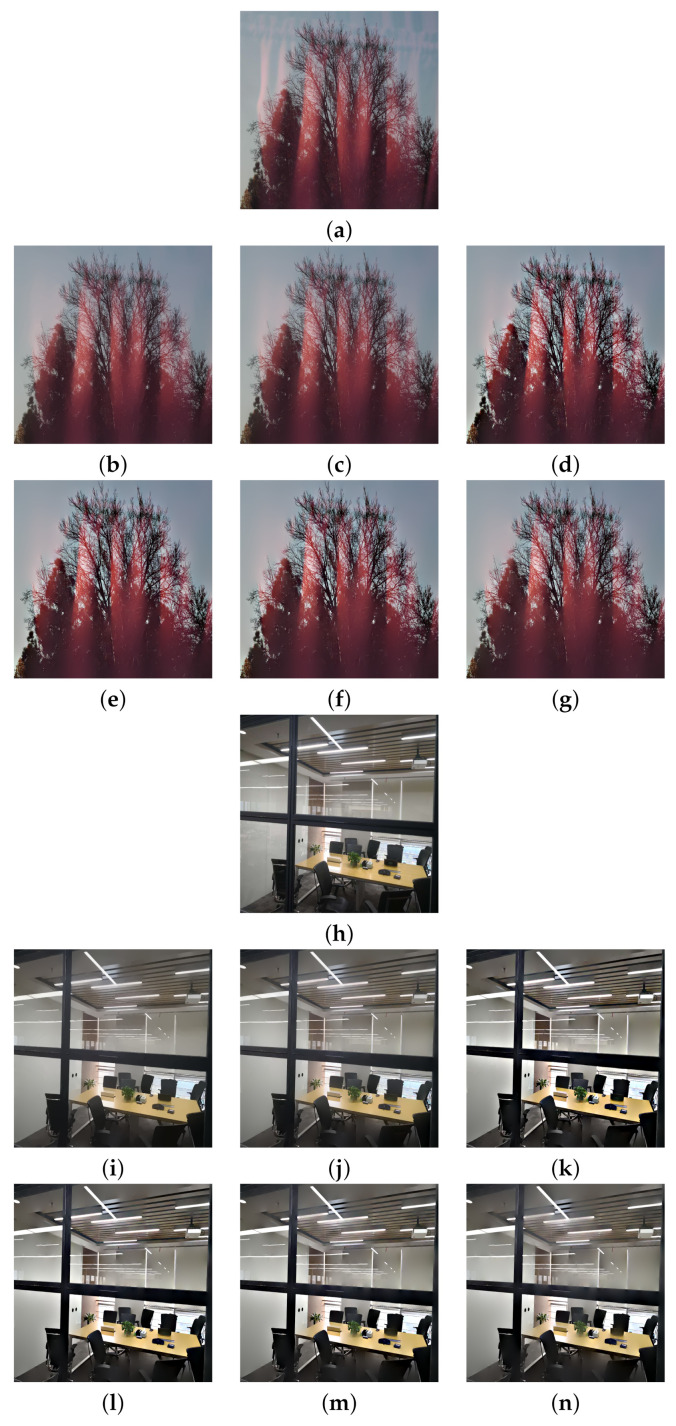
Reflection suppression results for real glass images ‘Tree’ and ‘Office’. (**a**,**h**) Input images. (**b**,**i**) The results of algorithm [2]. (**c**,**j**) The results of algorithm [3]. (**d**,**k**), (**e**,**l**), (**f**,**m**), and (**g**,**n**) are results of the proposed algorithm with N=1,2,3,4, respectively.

**Figure 8:**
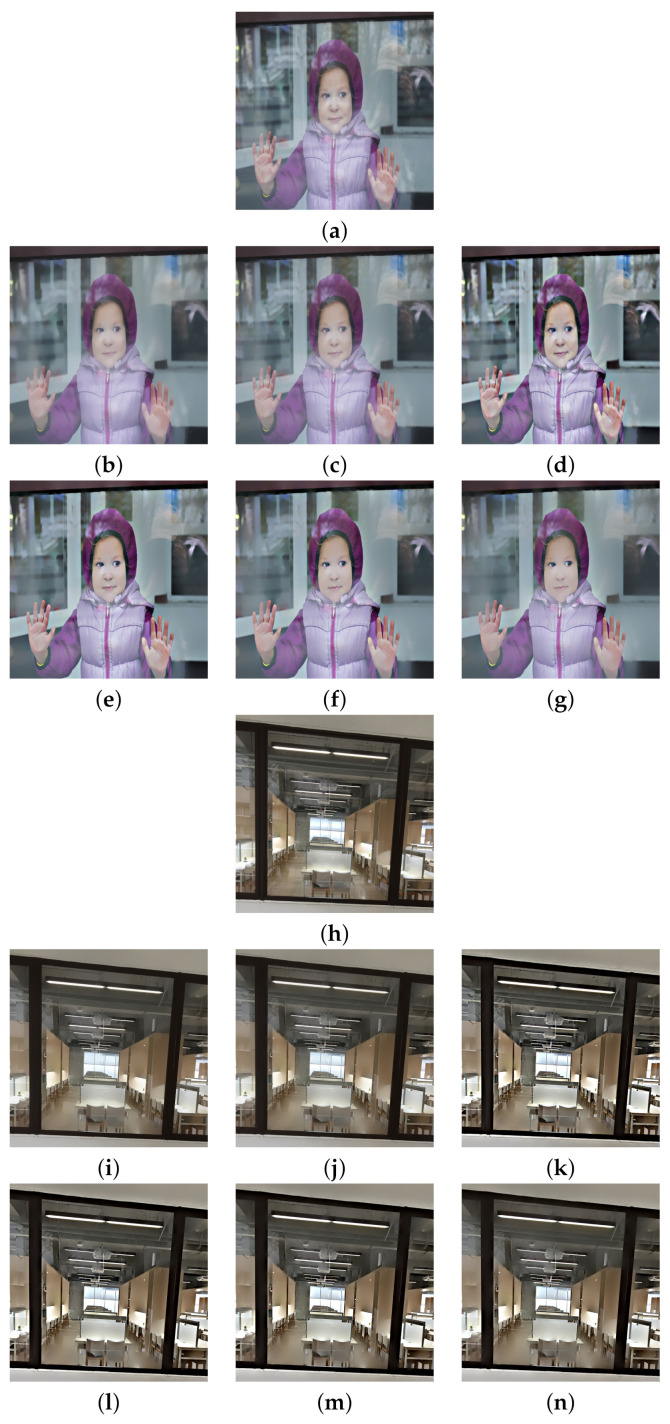
Reflection suppression results for real glass images ‘Child’ and ‘Library’. (**a**,**h**) Input images. (**b**,**i**) The results of algorithm [2]. (**c**,**j**) The results of algorithm [3]. (**d**,**k**), (**e**,**l**), (**f**,**m**), and (**g**,**n**) are results of the proposed algorithm with N=1,2,3,4, respectively.

The real glass dataset of Yang et al. [3] is used here. The test images were captured directly using various smartphones with different resolutions. Figure 6, Figure 7 and Figure 8 show the reflection suppression results of all the competing algorithms for the six real glass input images.

The ‘Dog’ image has some reflections on the window glass. Algorithms [2,3] smoothed out some of them, while the proposed algorithm suppressed all reflections and simultaneously enhanced image details. The ‘Girl’ image has a strong reflection on the left side such that all algorithms can not completely remove it. However, the proposed algorithm with N=4 suppresses the most reflections among all algorithms. The ‘Tree’ image has an extremely strong reflection that no algorithm can remove it. However, the proposed algorithm still has the best visual performance by enhancing the image contrast. The ‘Office’ image has many small rectangle shape reflections that are hard to suppress, the proposed algorithm with N=4 still suppresses most of them among all algorithms. The ‘Child’ and ‘Library’ images have many complex reflections and the proposed algorithm with N=4 still has the best visual performance. Here, we note that the images ‘Office’ and ‘Library’ are relatively challenging among all real examples. The main reason is that the rectangular-shaped window reflections have both bright intensities and sharp edges that violate the critical assumption that reflection gradients are smaller in magnitude and lower in focus compared to transmission gradients. If we want to completely remove those reflections, we need to set large *h* and we will lose many transmission edges as a heavy price. Therefore, the reflections of these two images are relatively hard to suppress. We further note that algorithm [3] and the proposed algorithm share the same model parameters *h* and ε for all input images.

Since we do not have reflection-free solutions for real glass images for computing PSNR values to quantitatively evaluate the algorithms, here, we compare their computation times instead. Table 2 shows the computation time in seconds for all six input images and all competing algorithms. Although the proposed algorithm costs much time than algorithm [3], it is still very efficient compared to algorithm [2].

### 4.3. Ablation Study

Here, we conduct an ablation study for the adaptive weight function φ used in the proposed model and provide an experiment of the proposed algorithm with various values of *h* and β.

**Figure 9:**
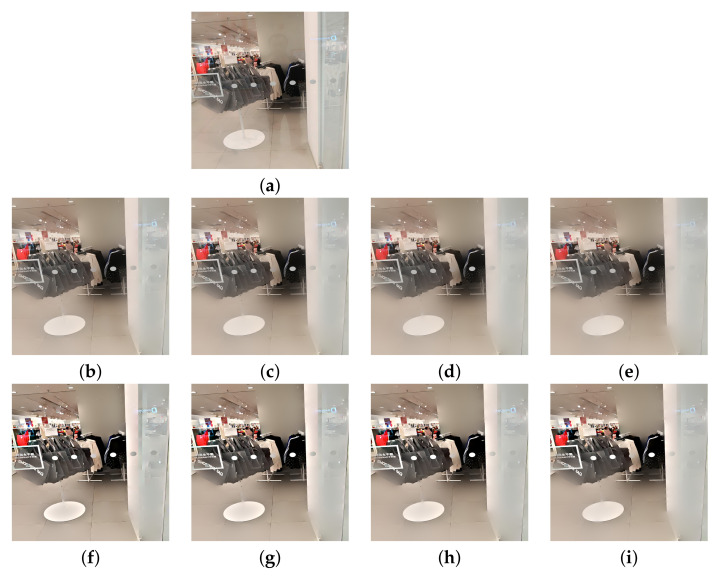
Reflection suppression results for real glass image ‘Mall’. (**a**) Input image. (**b**–**e**) The results of proposed algorithm (under φ=1) with N=1,2,3,4, respectively. (**f**–**i**) The results of algorithm (under φ in (12) and β=1) with N=1,2,3,4, respectively.

We begin with the ablation study by comparing the reflection suppression results of the proposed algorithm with and without the adaptive weight function φ, i.e., setting φ by (12) and by constant value 1, respectively. Experimental results are provided in Figure 9. From Figure 9b–e, one can observe that, under constant weight φ=1, more and more reflections are suppressed as *N* increases but the transmission edges also become increasingly blurred. Under the adaptive weight φ given in (12), weak transmission edges are enhanced and keep their sharpness as *N* increases. The results are shown in Figure 9f–i.

**Figure 10:**
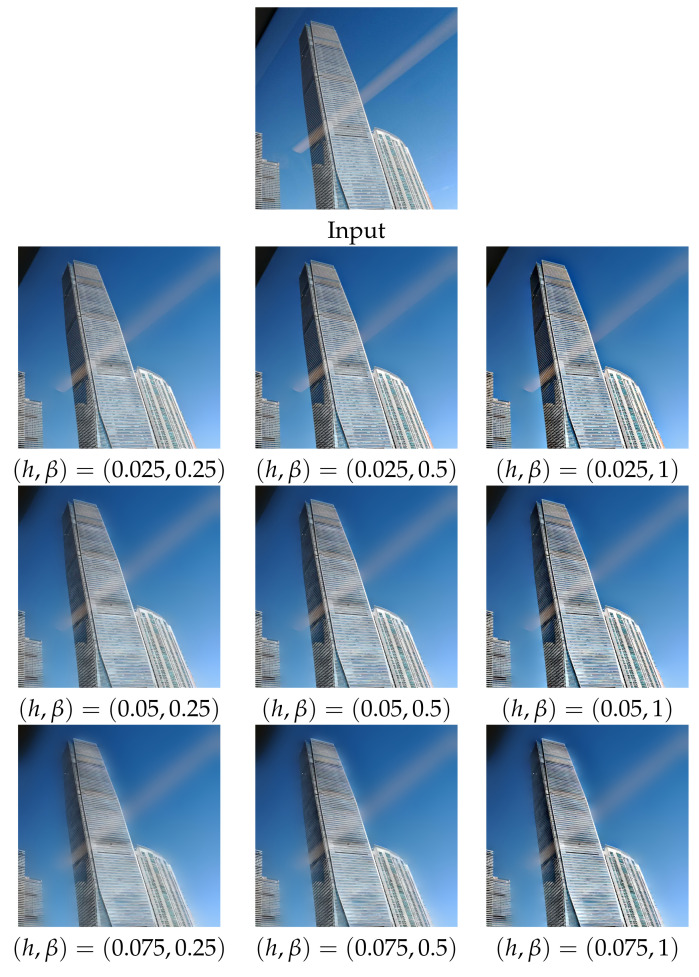
Reflection suppression results of the proposed algorithm (under N=2 and φ in (12)) for real glass image ’Building’ with various *h* and β.

To further elaborate the effects of parameters *h* and β to the proposed model, we test the proposed algorithm under N=2 and φ in (12) with h=0.025,0.05,0.075 and β=0.25,0.5,1. Experimental results are provided in Figure 10. It can be observed that, as the threshold parameter *h* increases, the strong reflections are increasingly suppressed but the weak transmission edges on the lower building also become increasingly blurred. However, as enhancement parameter β increases, those weak edges become increasingly enhanced. These two experiments demonstrate the role that adaptive weight function φ played in the proposed variational model.

## 5. Summary and Conclusions

In this paper, we have proposed a both effective and efficient method for suppressing image reflections. Unlike the previous methods that suppressed the reflections only in a single spatial scale, the proposed method can suppress both weak and strong and multiple scale reflections while preserving important edge information via a multiscale hard thresholding framework. A direct solver using the Fourier cosine transform for implementing the proposed method is also given. Experimental results demonstrate that the proposed new algorithm is able to achieve superior performance compared with the previous algorithms. Extending the multiscale idea used in our model for dealing with more challenging situations such as ghosting effect [15,26] should be interesting, and this deserves further studies.

## Figures and Tables

**Table 1:** PSNR values of the reflection suppression results in Figure 3, Figure 4 and Figure 5. The best value in each row is shown in boldface.

Image	Algorithm [2]	Algorithm [3]	Proposed (N=1)	Proposed (N=2)	Proposed (N=3)	Proposed (N=4)
Figure 3a	70.18	70.16	**70.73**	70.71	69.94	69.14
Figure 3h	66.36	66.37	66.87	**67.06**	66.82	66.39
Figure 4a	67.96	67.99	68.64	**68.65**	68.03	67.41
Figure 4h	63.96	63.97	64.31	**64.47**	64.27	63.96
Figure 5a	66.21	66.22	66.44	**66.68**	66.28	65.86
Figure 5h	62.07	62.07	62.15	**62.35**	62.24	62.05

**Table 2:** Computation time of the reflection suppression results in Figure 6. The best value in each row is shown in boldface.

Image	Size	Algorithm [2]	Algorithm [3]	Proposed (N=1)	Proposed (N=2)	Proposed (N=3)	Proposed (N=4)
Figure 6a	667 × 1000	445.3	**0.682**	0.775	0.941	1.062	1.246
Figure 6h	823 × 1200	661.2	**0.998**	1.145	1.353	1.617	1.785
Figure 7a	880 × 1193	699.8	**1.008**	1.158	1.402	1.666	1.884
Figure 7h	1080 × 1440	1036.6	**1.209**	1.388	1.734	2.159	2.472
Figure 8a	1131 × 1698	1377.9	**1.888**	2.042	2.536	2.987	3.492
Figure 8h	1992 × 2448	3234.2	**3.853**	4.401	5.476	6.501	7.934

## Data Availability

The algorithm performance is tested on the public datasets, including https://www.imageprocessingplace.com/downloads_V3/root_downloads/image_databases/standard_test_images.zip (accessed on 1 September 2021), https://data.mendeley.com/public-files/datasets/3hfzp6vwkm/files/68088adc-c463-417e-bd84-03804d2670ff/file_downloaded (accessed on 8 September 2021), and https://github.com/yyhz76/reflectSuppress/tree/master/figures (accessed on 15 September 2021).

## References

[1] Barrow H., Tenenbaum J., Hanson A., Riseman E. (1978). Recovering intrinsic scene characteristics. Comput. Vis. Syst..

[2] Arvanitopoulos N., Achanta R., Susstrunk S.T. Single image reflection suppression. Proceedings of the 2017 IEEE International Conference on Computer Vision and Pattern Recognition (CVPR).

[3] Yang Y., Ma W., Zheng Y., Cai J.F., Xu W. Fast single image reflection suppression via convex optimization. Proceedings of the 2019 IEEE International Conference on Computer Vision and Pattern Recognition (CVPR).

[4] Kong N., Tai Y.W., Shin J.S. (2008). A physically-based approach to reflection separation: From physical modeling to constrained optimization. IEEE Trans. Pattern Anal. Mach. Intell..

[5] Schechner Y.Y., Kiryati N., Shamir J. Blind recovery of transparent and semireflected scenes. Proceedings of the 2000 IEEE International Conference on Computer Vision and Pattern Recognition (CVPR).

[6] Farid H., Adelson E.H. Separating reflections and lighting using independent components analysis. Proceedings of the 1999 IEEE International Conference on Computer Vision and Pattern Recognition (CVPR).

[7] Agrawal A., Raskar R., Nayar S.K., Li Y. (2005). Removing photography artifacts using gradient projection and flash-exposure sampling. ACM Trans. Graph..

[8] Sirinukulwattana T., Choe G., Kweon I.S. Reflection removal using disparity and gradient-sparsity via smoothing algorithm. Proceedings of the 2015 IEEE International Conference on Image Processing (ICIP).

[9] Li Y., Brown M.S. Exploiting reflection change for automatic reflection removal. Proceedings of the 2015 IEEE International Conference on Computer Vision (ICCV).

[10] Xue T., Rubinstein M., Liu C., Freeman W.T. (2015). A computational approach for obstruction-free photography. ACM Trans. Graph..

[11] Gai K., Shi Z., Zhang C. (2012). Blind separation of superimposed moving images using image statistics. IEEE Trans. Pattern Anal. Mach. Intell..

[12] Guo X., Cao X., Ma Y. Robust separation of reflection from multiple images. Proceedings of the 2014 IEEE International Conference on Computer Vision and Pattern Recognition (CVPR).

[13] Levin A., Weiss Y. (2007). User assisted separation of reflections from a single image using a sparsity prior. IEEE Trans. Pattern Anal. Mach. Intell..

[14] Li Y., Brown M.S. Single image layer separation using relative smoothness. Proceedings of the 2014 IEEE International Conference on Computer Vision and Pattern Recognition (CVPR).

[15] Shih Y., Krishnan D., Durand F., Freeman W.T. Reflection removal using ghosting cues. Proceedings of the 2015 IEEE International Conference on Computer Vision and Pattern Recognition (CVPR).

[16] Wan R., Shi B., Hwee T.A., Kot A.C. Depth of field guided reflection removal. Proceedings of the 2016 IEEE International Conference on Image Processing (ICIP).

[17] Fan Q., Yang J., Hua G., Chen B., Wipf D. A generic deep architecture for single image reflection removal and image smoothing. Proceedings of the 2017 IEEE International Conference on Computer Vision (ICCV).

[18] Wan R., Shi B., Duan L.-Y., Tan A.-H., Kot A.C. Crrn: Multi-scale guided concurrent reflection removal network. Proceedings of the 2018 IEEE International Conference on Computer Vision and Pattern Recognition (CVPR).

[19] Yang J., Gong D., Liu L., Shi Q. Seeing deeply and bidirectionally: A deep learning approach for single image reflection removal. Proceedings of the 2018 European Conference on Computer Vision (ECCV).

[20] Zhang X., Ng R., Chen Q. Single image reflection separation with perceptual losses. Proceedings of the 2018 IEEE International Conference on Computer Vision and Pattern Recognition (CVPR).

[21] Schechner Y.Y., Kiryati N., Basri R. (2000). Separation of transparent layers using focus. Int. J. Comput. Vis..

[22] Xu L., Lu C., Xu Y., Jia J. (2011). Image smoothing via L0 gradient minimization. ACM Trans. Graph..

[23] Ma W., Morel J.-M., Osher S., Chien A. An L1-based variational model for retinex theory and its applications to medical images. Proceedings of the 2011 IEEE International Conference on Computer Vision and Pattern Recognition (CVPR).

[24] Ma W., Osher S. (2012). A TV Bregman iterative model of Retinex theory. Inverse Probl. Imaging.

[25] Hsieh P.-W., Shao P.-C., Yang S.-Y. (2020). Adaptive variational model for contrast enhancement of low-light images. SIAM J. Imaging Sci..

[26] Huang Y., Quan Y., Xu Y., Xu R., Ji H. (2019). Removing reflection from a single image with ghosting effect. IEEE Trans. Comput. Imaging.

